# Study of morphological variation of northern Neotropical Ariidae reveals conservatism despite macrohabitat transitions

**DOI:** 10.1186/s12862-018-1152-y

**Published:** 2018-03-27

**Authors:** Madlen Stange, Gabriel Aguirre-Fernández, Walter Salzburger, Marcelo R. Sánchez-Villagra

**Affiliations:** 1 Palaeontological Institute and Museum, University of Zurich, Karl-Schmid-Strasse 4, 8006 Zurich, Switzerland; 2 Zoological Institute, University of Basel, Vesalgasse 1, 4051 Basel, Switzerland

**Keywords:** Conservatism, Disparity, Fish, Geometric morphometrics, Morphological evolution, Phylogeny

## Abstract

**Background:**

Morphological convergence triggered by trophic adaptations is a common pattern in adaptive radiations. The study of shape variation in an evolutionary context is usually restricted to well-studied fish models. We take advantage of the recently revised systematics of New World Ariidae and investigate skull shape evolution in six genera of northern Neotropical Ariidae. They constitute a lineage that diversified in the marine habitat but repeatedly adapted to freshwater habitats. 3D geometric morphometrics was applied for the first time in catfish skulls and phylogenetically informed statistical analyses were performed to test for the impact of habitat on skull diversification after habitat transition in this lineage.

**Results:**

We found that skull shape is conserved throughout phylogeny. A morphospace analysis revealed that freshwater and marine species occupy extreme ends of the first principal component axis and that they exhibit similar Procrustes variances. Yet freshwater species occupy the smallest shape space compared to marine and brackish species (based on partial disparity), and marine and freshwater species have the largest Procrustes distance to each other. We observed a single case of shape convergence as derived from ‘C-metrics’, which cannot be explained by the occupation of the same habitat.

**Conclusions:**

Although Ariidae occupy such a broad spectrum of different habitats from sea to freshwater, the morphospace analysis and analyses of shape and co-variation with habitat in a phylogenetic context shows that conservatism dominates skull shape evolution among ariid genera.

**Electronic supplementary material:**

The online version of this article (10.1186/s12862-018-1152-y) contains supplementary material, which is available to authorized users.

## Background

Convergent evolution is common in adaptive radiations, including in three-spined sticklebacks [1–3], African Lake cichlids [4], Midas cichlids [5], or African barbs [6]. Morphological convergence that is triggered by ecological convergence is typically manifested in feeding-associated features such as the skull. Skull shape evolution has been studied in a variety of teleost fishes to determine the factors that influence evolutionary change. This has been done from two main perspectives: (i) one is a developmental perspective, by examining factors as modularity and integration. Morphological evolution is constrained by development and integration and can be enhanced by modularity [7] but this is not a universal pattern [8]. With respect to teleost fishes, several recent studies investigated whether or not integration or modularity facilitate radiation [9–12]. The other approach (ii) examines species diversification from an adaptational perspective by investigating factors such as predator avoidance, niche occupation, or ecological functioning. In this context, previous studies either focused on the biomechanical link of skull or mandible shape to functional ecology [13–17] or explicitly investigated convergent evolution of skull shape and biotic and abiotic covariates [6, 16, 18, 19]. Other studies are exploratory or descriptive in nature [20–22]. Some of the above mentioned studies revealed that species that inhabit the same ecological niche converge in shape [9, 13–15, 17, 18]. This seems to be a common pattern also among terrestrial vertebrates [23–26], although mismatches of form and function triggered by behavioural plasticity and diverse constraints exist as well [27]. In terrestrial vertebrates the impact of phylogenetic dependence on shape similarity among closely related taxa has been tested explicitly [26, 28, 29] and several studies have examined these in teleost fishes, as well, using individual adaptive traits for ecological niches [30–32].

Previous studies that have investigated skull shape diversity and evolution in teleost fishes either applied traditional morphometric approaches based on linear measurements [13, 14, 33], or — more commonly — landmark-based two-dimensional geometric morphometrics (2D GM) [6, 10, 11, 15–17, 20–22, 34]. Currently, there is only a single study on fish skull shape evolution that applied more sophisticated three-dimensional geometric morphometric analyses (3D GM) [9].

In this study we aimed to add to the spectrum of methods that are applied to study teleost skull shape diversity and to fill an “organismal gap”. Firstly, we focus on variation of skull shape in a teleost group understudied with respect to their natural history, Ariidae (sensu [35]). By doing so, we aimed at exploring shape variation of ariid species from marine, brackish, and freshwater habitat from a defined geographic area. In an earlier study, this species assemblage has been shown to exhibit habitat-specific opercle bone shapes [36]. We aimed to follow the question whether the skull exhibits a similar pattern of shape adaptation. Secondly, we combine the study of shape variation of a composite adaptive trait, the teleost skull, using 3D GM, representing the second study only to use this method in teleosts. We consider the analysis of three-dimensional shape being advantageous as it is more accurate to capture shape information compared to 2D GM [37]. Thirdly, we combine the study of shape variation with a test for the influence of phylogenetic dependence and take that dependence into account while analysing co-variation with habitat.

Previous studies in fishes that investigated how and in which direction body shapes change after habitat transition have been performed in sticklebacks but these led to inconsistent results. Most studies find that marine populations are deep-bodied [38–40], have smaller eyes [38], and are larger compared to their freshwater sister taxa [41, 42]. This is contrasted by studies that find freshwater populations to be deep-bodied [42] and to have smaller eyes [43] and shorter heads [44]. The contrasting patterns that occur in marine-freshwater transitions in stickleback are summarized in [45]. Based on these previous findings we cannot hypothesize what changes are to be expected after habitat transitions — but that changes are to be expected.

We analysed skull shape variation in 28 species representing six genera (*Ariopsis*, *Bagre*, *Cathorops*, *Notarius*, *Potamarius*, and *Sciades*) of northern Neotropical Ariidae (subfamily Ariinae [35]) from marine, brackish, and freshwater habitats. Ariidae are widely distributed in all tropical and subtropical marine regions, as well as in near-coastal rivers and lakes. Freshwater environments are inhabited by species that adapted secondarily to freshwater [46] during independent habitat transitions [47]. Only 4% of ray-finned fishes manage to live in both marine and freshwater [48]. The evolutionary history of habitat transitions from freshwater (ariid ancestors) to marine and back to freshwater, with the availability of intermediate species with brackish occurrence, makes the Ariidae a valid system to study marine-freshwater transitions and associated shape changes. We categorise the ecological co-variate in macrohabitats (marine, brackish, and freshwater) as a collective proxy for differences in their ecological niche. Very little is known about the natural history of the individual species, e.g. feeding preferences, migration behaviour during breeding season, number of growth cycles per year, age at maturity, or longevity. We focused on the geographically circumvented taxa of the northern Neotropics in order to concentrate our resources and to appropriately identify the habitat that the species and different populations of the same species occupy. We make use of a new time-calibrated phylogenetic hypothesis of northern Neotropical Ariidae that was inferred by Bayesian inference from single-nucleotide polymorphism (SNP) markers, which also includes two newly discovered cryptic species [49], one of them with a different habitat occupation than its sister species, which enriches our analysis of shape variation.

We hypothesized that skull shape in northern Neotropical Ariidae diverges when species transit from the marine to the freshwater habitat, driven by e.g., differences in osmoregulation or biotic resources that affect freshwater, brackish, and marine living species differently. Secondly, we test whether shapes within each habitat converge due to the same reasons as described above.

## Methods

### Collection of specimens, sample sizes, and definition of grouping factors used in statistical analyses

**Fig. 1:**
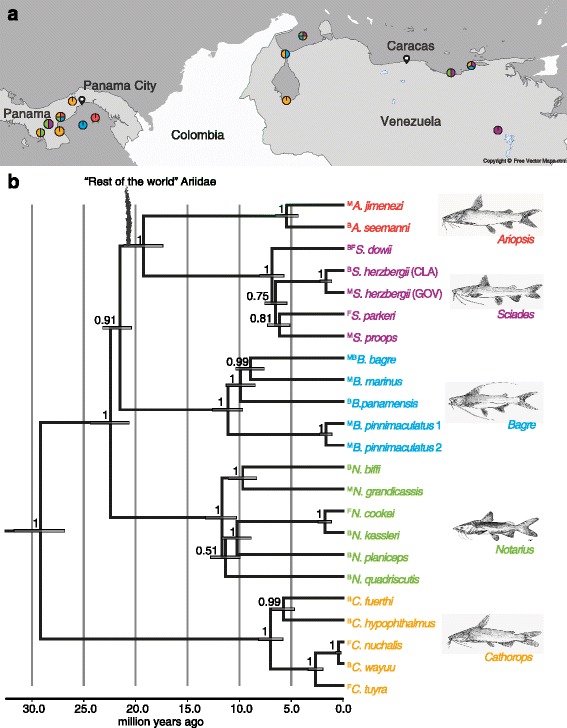
Geographic locations of sampling sites and phylogenetic relationships of sampled ariid species. **a** Map excerpt of the Northern Neotropics focusing on Panama and Venezuela. Positions of the filled circles indicate the sampling sites, details can be found in Additional file 1: Table S2. Sampled genera at each location are indicated by the colour of the filled circles. Colour key follows tip label colour in panel (**b**). **b** Bayesian inference of phylogenetic relationships within Northern Neotropical Ariidae. Phylogeny modified after Stange et al. [49] and used for phylogenetically informed shape analyses. Bayesian posterior probability (BPP) values for topology were calculated from 1000 trees. Grey bars represent 95% height probability distribution (HPD). The split of the “rest of the world” Ariidae is indicated in the topology for clarification of phylogenetic relatedness to geographically distant members of the ariid family. Other American genera that were not sampled here would appear nested in the presented tree

Specimens from Northern Neotropical ariid species (Additional file 1: Table S1) that occur in marine, brackish, and freshwater habitat were collected at the Caribbean coast of Venezuela and the Eastern Pacific coast of Panama (TEP) (Fig. 1a). Fishes were bought dead but fresh from local fishers. Fishes in Panama were collected under research and collecting permit no 59 (2014-2015) and exported under permit no. 65 granted by Autoridad de los Recursos Acuáticos de Panamá, Dirección General de Ordenación y Manejo integral (ARAP); and in Venezuela under research and collecting permit no. 001 (2014-2015) granted by Gobierno Bolivariano de Venezuela, Ministerio del Poder Popular para la Agricultura y Tierras, Instituto Socialista de la Pesca y Acuicultura. Specimens were measured and photographed, and skulls were macerated and bleached in the field. In addition 19 museum specimens (n(m) = 1; n(b) = 3, n(f) = 15) of the genera *Potamarius* (two species), *Ariopsis* (two species), and *Cathorops* (one species) were included (see Additional file 1: Table S3 for details) to extend sampling of freshwater species that we could not cover by our own fieldwork. We sampled eleven specimens of *Doraops zuloagai* (Doradidae), an obligate freshwater species, for morphological comparison with a related outgroup. Due to the currently unresolved higher level phylogeny of Siluriformes we cannot identify the sister taxon of Ariidae [50]. Note that the species identity of the specimens collected by us has been validated in a previous study using a barcoding approach [36], and a phylogenetic hypothesis based on 1768 bi-allelic single-nucleotide polymorphisms (SNPs) derived from restriction-site associated DNA-sequencing (RAD-seq) is available as well [49] and summarized in Fig. 1b.

For the investigation of morphological similarity in skull shape of specimens from similar habitat within Ariidae and comparison to one freshwater species from another siluriform family, we analysed, in total, 270 specimens (Additional file 1: Tables S1 and S3) of *Ariopsis* (A, *n* = 23), *Bagre* (B, *n* = 50), *Cathorops* (C, *n* = 73)*, Notarius* (N, *n* = 35), *Sciades* (S, *n* = 70), *Potamarius* (P, *n* = 8), and finally *Doraops* (D, *n* = 11) as outgroup, all in all 27 recognised and two cryptic species. Sample sizes per habitat were 125 from marine, 91 from brackish, and 54 from freshwater habitat.

A sub-dataset was generated for phylogenetically informed analyses containing 240 ariid specimens (Additional file 1: Table S1) without outgroup and museum specimens, containing 124 marine, 88 brackish, and 28 freshwater specimens.

### 3D geometric morphometrics analysis

#### Landmarks

**Fig. 2:**
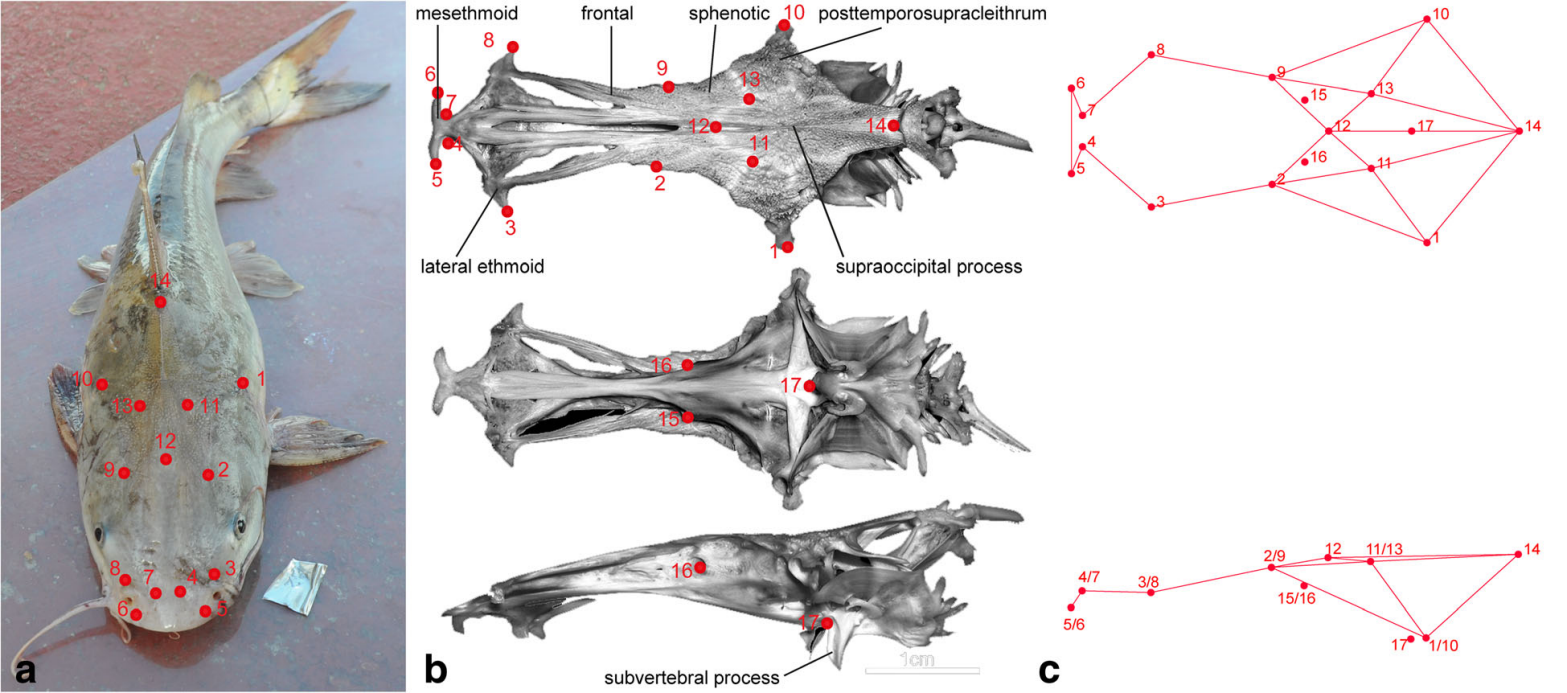
Positions of the seventeen landmarks that were used to study neurocranial shape change. For definitions see Additional file 1: Table S4. **a** specimen of the genus *Cathorops* with landmarks superimposed (photo credit: Madlen Stange); (**b**) neurocranium (*Cathorops arenatus* [photo credit: Catfish Bones – The Digital Atlas Of Catfish Morphology, http://catfishbone.ansp.org/]) in dorsal, ventral, and lateral view with numbered landmarks and relevant skeletal structures named; (**c**) wireframe connecting the landmarks, that are used for illustration of shape changes

Landmarks (LM) are defined as homologous points on which explanations of biological processes are based upon [51]. We collected eight Type 1 landmarks (discrete juxtapositions of tissues, here of bones) plus nine Type 2 landmarks (maxima of curvature and extreme points) (Additional file 1: Table S4) on the dorsal and ventral side of the neurocranium as illustrated in Fig. 2. Landmarks four to seven capture the shape of the mesethmoid, which is where the maxillary teeth are attached and the olfactory and other sensory nerves exit; landmarks three and eight mark the most distal points of the ethmoid, capturing the maximal extension of the anterior part of the neurocranium (here, the vomerine teeth are attached); landmarks two and nine represent the meeting point of the sphenotic and the frontal and capture more or less the narrowest part of the neurocranium; landmarks one and ten describe the most distal points and capture the maximal extension of the posterior part of the neurocranium (or posttemporosupracleithrum); landmarks 11 and 14 outline the supraoccipital process, the exterior roof of the posterior portion of the braincase. Landmark positions were measured in the laboratory using a MicroScribe ™ G2 with an accuracy of 0.38 mm. This portable device measures coordinates in 3D space and provides x, y, and z coordinates in a text-file. To record 3D landmarks, the skulls were mounted vertically on plasticine attached to the vertebral column, allowing measurements on the ventral and dorsal side without rotating the specimen. To assess the relative measurement error, two replicates for a subset of the specimens (*N* = 15) were taken and analysed using Procrustes analyses of variance (ANOVA) (see below). The effect of interspecific variation was larger (*F* = 31.40, *P* < 0.0001) than the variation between measurements of the same specimen (*F* = 0.58, *P =* 1). Therefore, we measured each specimen once for the final geometric morphometric analysis.

#### Procrustes superimposition

All geometric morphometric analyses were carried out in the R package geomorph v.3.0.5 [52] in R v.3.3.3 [53] unless stated otherwise. Missing landmarks (one to two individuals with one to two missing LMs per species set, 15 species were affected), caused by minimally broken skulls, were extrapolated using the function *estimate.missing* based on the thin-plate spline (TPS). Generalized Procrustes superimposition (GPA) [54] of all measured neurocrania was performed in order to remove the effects of size, orientation and position. GPA was performed accounting for object symmetry and only the symmetric component was used for subsequent shape analyses.

### Analyses of shape variation of Neotropical marine and freshwater catfish species

Principal component analysis (PCA) (*plotTangentSpace*) was applied in order to visualize morphospace occupation and to reduce dimensionality of the shape data to identify major axes of variance. PC1-PC2 morphospace plots visually aid to identify patterns of clustering and related skull shape changes. We highlighted the occupied PC1-PC2 morphospace for each habitat (fresh, brackish, and sea water) and genus (Ariidae: *Ariopsis*, *Bagre*, *Cathorops*, *Notarius*, *Potamarius*, *Sciades*; outgroup — Doradidae: *Doraops*) by convex hulls.

To assess whether shapes differ among habitats, Procrustes ANOVA (*procD.lm*) with 1000 random permutations of the residuals among groups for significance testing was applied. To visualize the differences in shape among the habitats the group mean shape for habitats within Ariidae were calculated and compared to the mean shape of the freshwater outgroup. The Procrustes distances among the groups were calculated from the square root of the sum of squared differences in all landmark coordinates between the group mean shapes of any group combinations.

We quantified the extent of morphospace occupation of 26 recognised and two cryptic ariid species (Additional file 1: Tables S1 and S3). First, we quantified overall disparity (MD) (*morphol.disparity*), the space all analysed specimens occupy in morphospace, by calculation of the grand mean or centroid (shape~ 1) in unit Procrustes variance. Further, the contribution of each habitat group and each genus to overall disparity was calculated by inferring Foote’s partial disparity (PD) [55]. To do so, residuals obtained from the overall mean were used and the squared residual lengths were summed over either group mean (shape~ 1, groups = ~genus or groups = ~habitat). The resulting group-wise Procrustes variances were multiplied by number of samples per group (n) divided by total sample size (259) minus one. By this procedure the partial disparity of each group sums up to the overall disparity of the entire dataset and assertions about the percental contribution of each group to the overall disparity of all analysed specimens can be made.

### Phylogenetically informed analyses

To investigate the influence of habitat and phylogeny on skull shape variation we performed analyses that take the phylogenetic relatedness into account. The following analyses were carried out on a subset of the shape dataset (Additional file 1: Table S1) that contains only species that are present in the available phylogeny. To this end, we first calculated mean shapes per species using the *mshape* function. The phylogenetic tree (Fig. 1 b) was taken from Stange et al. [49]. The tree is derived from a multispecies-coalescent analysis based on single-nucleotide polymorphisms and internal node calibration based on fossils instead of the common biogeographic calibration point, the final closure of the Panamanian Isthmus. It was read in using *read.nexus* (ape).

Blomberg’s *K* [56] is an estimator that assesses the strength of phylogenetic signal in any quantitative variable. Phylogenetic signal in this context is the association of phenotypic similarity derived from Procrustes coordinates to phylogenetic relatedness among the taxa under study and is determined by the generalized version of *K* for multivariate data [57]. The estimation of *K* is implemented in the *physignal* function, which was run on the averaged species shape data with 1000 random permutations for significance testing. *K* is the ratio of the observed trait variance and the expected trait variance as predicted under Brownian motion. *K* has an expected value of 1 under Brownian motion (strong phylogenetic signal), a *K* < 1 implies higher shape divergence of taxa, and a *K* > 1 implies more shape similarity of closely related taxa than expected by a Brownian motion model of trait evolution. *K* = 0 resembles the null hypothesis, stating that there is no phylogenetic signal in the shape data and that closely related taxa are not more similar to each other than distantly related taxa [57].

To test whether the distinctiveness of habitat-specific shapes holds true also after taking the phylogenetic dependence of the taxa into account, we performed a phylogenetic ANOVA on the shape data (Procrustes coordinates). The shape data were analysed applying a generalized least squares approach [58, 59], as implemented in the *procD.pgls* function. The significance of differences among groups was tested in a permutation test based on residual randomization [60] (RRPP = TRUE) with 999 random permutations.

We assessed phenotypic convergence, hypothesizing that species from the same habitat group would ‘converge’ towards similar shapes. First, for an initial visual inspection, we produced a phylomorphospace plot in PC1-PC2 shape space. The phylogeny was projected on the mean species shape scores of the tip data and the reconstructed ancestral states derived from maximum likelihood analysis using the *plotGMPhyloMorphoSpace* function. Second, following the argumentation by Zelditch et al. [29] we tested for convergence in the full shape space instead of using principal components, as the latter do not exhibit independent rates of adaptation and diffusion. We chose to apply the ‘C-metrics’ [61] as these are also applicable to multi-dimensional shape data opposed to SURFACE [62] which is only suitable for multivariate data [29]. We therefore follow the procedure proposed by Zelditch et al. [29] to first compute a tanglegram using the *cophylo* function from phytools [63], comparing the phylogeny and the phenogram. The phenogram is a UPGMA tree computed from Procrustes distances from species mean configurations. Lines are drawn between the phylogeny and the phenogram connecting identical tips. Convergence is indicated by crossing lines in the tanglegram and those instances are chosen to be analysed with the ‘C-metrics’. We calculate C1 to C4 by using the *calcConv* function as provided in the supporting information of Zelditch et al. [29]. This code has been adapted to perform calculations based on distances in the full shape space instead of being based on principal components. C1 measures the distance in shape space of two species as a proportion of the maximum distance the lineages have experienced. C2 is based on the same distance measures as C1 but it is measured on an absolute scale in contrast to being relative to the maximum phenotypic distance. C1 and C2 are comparable within datasets but not between them. C3 and C4 are based on standardising C2 for the total amount of evolutionary change leading from the most recent common ancestor (MRCA) to both tips, and standardising by the total amount of evolutionary change along all lineages descended from the MRCA of the two focal tips, respectively, which allows comparison between data sets [61, 64]. We do not analyse C5 here (the frequency of convergence) as the dataset does not contain more instances of convergence than variables.

## Results

### Morphospace occupation, morphological disparity, and quantification of shape differences

**Fig. 3:**
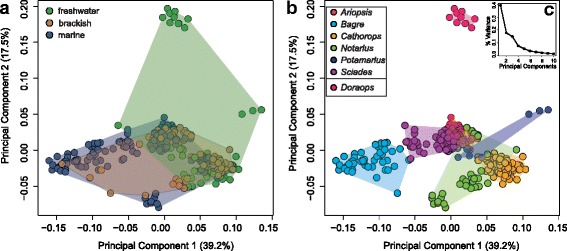
Morphospace occupation of 259 analysed ariid individuals (28 species) and 11 freshwater outgroup specimens of the genus *Doraops* (Doradidae) along principal component axes one and two. Specimens are highlighted by (**a**) habitat and (**b**) genus. Convex hulls highlight the margins each group occupies in morphospace. **c** Screeplot of principal components. The biggest variance is covered by the first two PCs, which account for 56.7% of the observed variation

To investigate whether species that live in a similar habitat evolved similar phenotypes – here assessed by skull shape – we inferred the shape space that is occupied by 28 Neotropical ariid and one doradoid species (270 individuals, 7 genera) living in marine, brackish, and freshwater habitats (Fig. 3). The first two principal components describe 56.7% of the observed overall shape variation (Fig. 3 C). Visual inspection of the PC1-PC2 shape space (Fig. 3 a) revealed that freshwater species, ariid and doradoid, are mostly situated at positive PC1 values, whereas brackish and marine species occupy almost the entire range of PC1. PC2 separates ariid species from the doradoid species. When the same scatterplot is coloured by genus, we see that species that belong to the same genus cluster together and mostly overlap to a certain degree, and that only *Bagre* — a pure marine genus — in its full shape range occupies an individual shape space at negative PC1 values opposite of freshwater species.

**Fig. 4:**
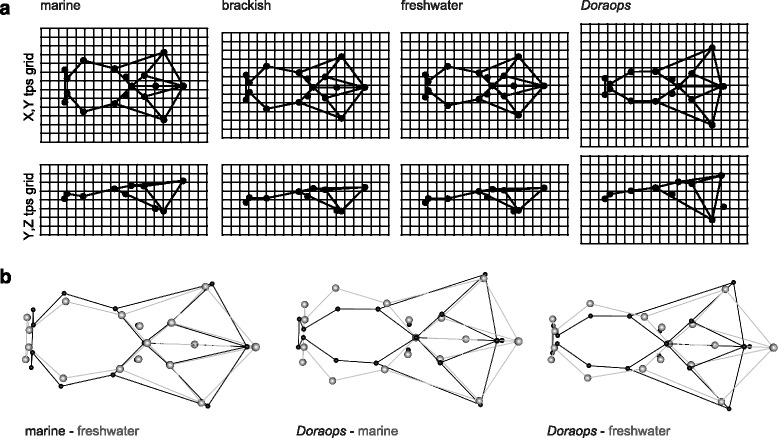
Skull shape changes from marine to freshwater as point illustrations with lines connecting landmarks (for your guidance see Fig. 2). **a** Procrustes mean shapes for marine, brackish, and freshwater ariids, and freshwater *Doraops zuloagai*. A common feature shared by freshwater ariids and the freshwater doradoid as compared to marine Ariidae is the very narrow mesethmoid. Within Ariidae when comparing marine with freshwater specimens the neurocranium elongates, becomes narrower and flatter. **b** Shape changes visualised by superimposition of mean shapes of, from left to right, marine–freshwater ariids, marine ariids–*Doraops*, and freshwater ariids–*Doraops*. Within Ariidae, the proportions of the anterior and posterior part of the neurocranium change to a longer and narrower frontal part and a smaller and flatter braincase. *Doraops* and freshwater ariids have about the same length, but exhibit an even narrower frontal part, and an even more distinct posterior part

We tested for shape differentiation among habitat in the entire Procrustes shape space and find that marine, brackish, and freshwater ariid species are significantly different from each other (Z = 6.996, *P* = 0.001) with marine and freshwater species having the largest Procrustes distance from each other (0.0961, Table 1). We did not test differentiation with *Doraops* as this will mostly be driven by phylogenetic distance. Morphological changes that are associated with marine-freshwater transition are narrower lateral ethmoids and mesethmoids exhibited in both freshwater ariid species and the freshwater doradoid species (Fig. 4). Due to the clustering by genus in PC1-PC2 shape space we tested whether shapes of genera differ in overall shape space. We find significantly different shapes among genera (Z = 14.887, *P* = 0.001) and the most distinct from each other are *Bagre*, a pure marine genus, and *Potamarius*, a pure freshwater genus (Table 2). The Procrustes distance of the freshwater doradoid species to any of the ariid habitat groups is about 0.2; the distance to ariid genera is smallest to *Ariopsis* (0.18) and largest to *Bagre* (0.24). The Procrustes distance to the pure freshwater genus, *Potamarius*, is comparably large with 0.21.

**Table 1:** Procrustes variance and Procrustes distances for habitat groups, all ariid genera

	fresh	brackish	marine
Procrustes variances	0.009127	0.007463	0.010751
Partial Disparity	0.002089 18.92%	0.002950 26.73%	0.006000 54.35%
Procrustes distance			
fresh	0		
brackish	0.0348	0	
marine	0.0961	0.0681	0

**Table 2:** Procrustes variance and Procrustes distances for ariid genera

	*Ariopsis*	*Bagre*	*Cathorops*	*Notarius*	*Potamarius*	*Sciades*
Procrustes variance	0.002619	0.004829	0.002915	0.004904	0.006997	0.005364
Partial disparity	0.000465 4.21%	0.003707 33.58%	0.002844 25.77%	0.001044 9.45%	0.000584 5.29	0.002395 21.70%
Procrustes distance						
*Ariopsis*	0					
*Bagre*	0.1423	0				
*Cathorops*	0.0966	0.1890	0			
*Notarius*	0.0754	0.1511	0.0853	0		
*Potamarius*	0.0978	**0.2015**	0.1134	0.1048		
*Sciades*	**0.0646**	0.1354	0.1217	0.0783	0.1281	0

In bold the smallest and the largest distance

The overall disparity (MD), i.e. the shape (Procrustes) variance of ariid specimens, is 0.011. The partial disparity (PD) of ariid species that live in a specific habitat contribute to MD is highest for marine and lowest for freshwater species (summarized in Table 1). This pattern does not change when the genera with obligate marine or freshwater species (*Bagre*, *Potamarius*) were excluded from the analyses (Table 3). The PD that each genus contributes to this total variance is smallest in *Ariopsis* (0.0005, 4.2% of the total variance), and largest in *Bagre* (0.0037, 33.6%). The genus-specific absolute variance was smallest in *Ariopsis* and largest in *Potamarius* (Table 1). The difference in PD and absolute variance is that the residuals of the latter were obtained from the group mean and not from the overall mean. Therefore, absolute variance informs on the expansion in morphospace unrelated to the other genera and sample size.

**Table 3:** Procrustes variance and Procrustes distances for habitat groups based on genera with species in different habitats (*Ariopsis*, *Cathorops*. *Sciades*, *Notarius*). *Potamarius* and *Bagre* were excluded as they are restricted to a specific habitat

	fresh	brackish	marine
Procrustes variances	0.007630	0.006408	0.007776
Partial Disparity	0.001256 20.44%	0.002229 36.27%	0.002660 43.28%
Procrustes distance			
fresh	0		
brackish	0.0311	0	
marine	0.0652	0.0413	0

### Phylogenetically informed analyses reveal conservatism in neurocranial shape

**Fig. 5:**
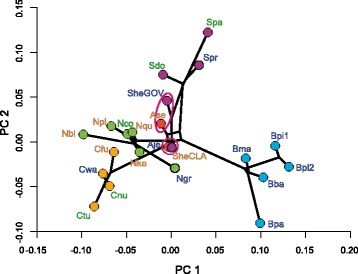
Phylomorphopsace plot from the first two major axes of variance derived from 3D GM skull shape data and the RAD-seq derived phylogeny. Colour of the filled circles indicate genus affinity, colour of tip labels indicate habitat affinity as defined in Fig. 3. Cases of possible shape convergence, further supported by the tanglegram (Fig. 6) and analysed in respect to strength of convergence using the ‘C-metrics’, are indicated with pink ellipses

**Fig. 6:**
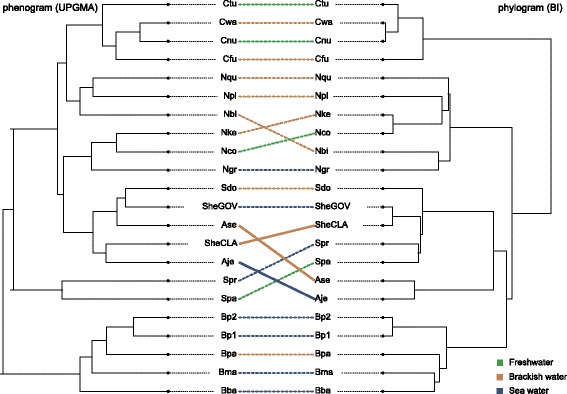
Tanglegram of morphological and genetic distance trees. Crossing lines are an indicator of incongruence of morphology and phylogeny. Cases of possible convergence are indicated with solid lines and were further analysed using the ‘C-metrics’

An estimator of phylogenetic signal suggests strong phylogenetic signal in the overall shape data (K = 0.6896, *P* = 0.001). A phylogenetic ANOVA based on the overall shape data finds no significant differences among shapes from different habitats (Z = − 2.6102, *P* = 0.993). To visualize these results we have reconstructed the phylomorphospace (Fig. 5), which also demonstrates little evidence for convergent evolution of species from similar habitats, as species from the same habitat do not occupy similar spaces in phylomorphospace and lines rarely cross. Yet two cases of convergence occur, namely *A. seemanni* (Pacific, brackish) and *S. herzbergii* GOP (Gulf of Venezuela, marine), and *A. jimenzi* (Pacific, marine) and *S. herzbergii* CLA (Clarines, Venezuela, Caribbean, brackish) (Fig. 5). The same instances of convergence become apparent in the tanglegram of pheno- and phylogram (Fig. 6). Interestingly, those converging species do not occupy the same habitat, but brackish and marine habitat, each, and are also found in different oceans, the Tropical Eastern Pacific and Caribbean. The observed cases of convergence are also larger than expected by chance (Table 4, C1). Both possibly convergent pairs show (*A. jimenzi*, *S. herzbergii* CLA and *A. seemanni* and *S. herzbergii* GOV) show 30-32% convergence (C1), which represents 14-11% (C3) of the total evolution of those lineages and 0.6-0.5% (C4) of the total evolution in the clade containing those taxa (Table 4). C2 takes into account the magnitude of change that the two taxa had to accomplish. The small C2 (0.023 and 0.018) compared to the large C1 (0.305 and 0.324) indicates that those two taxa are very similar in the first place. The convergence of *A. seemanni* and *S. herzbergii* GOV is not significant according to C2-C4 (Table 4).

**Table 4:** Strength of convergent evolution based on the’ C-metrics’ including the calculated probability that the observed convergence exceeds what would be expected from randomly evolving lineages (*P*), and Procrustes distance (ProcD) between convergent taxa in Procrustes shape space

convergent taxa	C1	C2	C3 (= C2/L_tot.lineage_)	C4 (= C2/L_tot.clade_)	ProcD
*A. jimenzi* + *S. herzbergii* CLA	0.305 (*P* = 0.038)	0.023 (*P* = 0.041)	0.137 (*P* = 0.067)	0.006 (*P* = 0.061)	0.065
*A. seemanni* + *S. herzbergii* GOV	0.324 (*P* = 0.023)	0.018 (*P* = 0.087)	0.113 (*P* = 0.107)	0.005 (*P* = 0.108)	0.049

L_tot.lineage,_ L_tot.clade_ are defined as the total amount of change from the common ancestor of the convergent taxa leading to those taxa, and of the entire clade that contains the convergent taxa, respectively (see [61] for details). Values of C3 and C4 can be compared across datasets, whereas C1 and C2 can only be compared within the presented study

## Discussion

We explored the variation of skull shape in a species-rich radiation of teleost fishes, namely Ariidae (sea catfishes), that occupy a huge range of salinity regimes, from salt to freshwater, with freshwater being the derived habitat. Further, we investigated whether species from the same habitat evolved similar skull shapes, comparing also to a siluriform freshwater relative, a doradoid species (*Doraops*). The only morphological feature that freshwater ariids shared with the freshwater outgroup species was the narrow and longer snout. Also, the marine species are more deep-bodied than the freshwater species as derived from the maximum skull height, which can be taken as a proxy for overall body height. This supports some previous findings in sticklebacks that freshwater species are more shallow-bodied than their marine ancestors [38–40].

Our results demonstrate that skull shape variation underlies phylogenetic conservatism and only one significant case of convergence, between *A. jimenzi* and *S. herzbergii* CLA, occurred. However, this case was not based on convergence due to habitat occupation as the former occurs in marine and the latter in brackish habitat. A possible explanation that needs to be tested is ecomorphological convergence occurring in the microscale in these species, e.g. type of bait. *A. jimenzi* has just been described [65] and *S. herzbergii* CLA is an undescribed cryptic species [36, 49], therefore we cannot elaborate on their natural history but we can only point out how little we know about this particular clade of teleost fishes.

We found significant differences in skull shape among habitats when we did not correct for the phylogenetic dependence of the species. When shape differences among species were accounted for their phylogenetic relationships this signal vanished, highlighting the importance of taking the phylogenetic relatedness in analyses of co-variance into account.

The exclusion of the obligate marine genus *Bagre* and the obligate freshwater genus *Potamarius* enabled an unbiased view on morphospace that is occupied by genera with species in all three habitats. Marine and freshwater species retained similar variances but the PD of marine species reduced due to the exclusion of *Bagre*. This demonstrates that the general pattern of marine species occupying the largest morphospace, although insignificantly different from freshwater species, holds true. The restriction in the ability to expand in morphospace seems to lie rather on the brackish than freshwater species. This might indicate a constraint that is put on species that cope with two environmental regimes.

## Conclusions

The combination of 3D geometric morphometrics with a solid phylogenetic hypothesis for northern Neotropical Ariidae aided to identify patterns of skull shape diversification. We found that skull shape is mostly determined by phylogeny and only a single case of convergence in shape occurred, yet, this could not be explained by our habitat covariate. Freshwater species occupy the smallest place in shape space compared to brackish and marine species and differ most from marine species, possibly caused by their young clade age or competition in their new habitats.

## Additional file

Additional file 1: Supporting information for the Methods and Results section. Contains **Tables S1** to **S4**: list of species and number of individuals used in this study, geographic locations, and definitions of geometric morphometric landmarks, and **Figure S1** – PC scatterplot with species highlighted. (DOCX 261 kb)

## Abbreviations

2D: Two-dimensional
3D: Three-dimensional
A: *Ariopsis*
ANOVA: Analyses of variance
B: *Bagre*
b: Brackish
C: *Cathorops*
CLA: Clarines (a locality information)
D: *Doraops*
f: Freshwater
GM: Geometric morphometrics
GOP: Gulf of Venezuela
GPA: Generalized Procrustes superimposition
LM: Landmark
m: Marine
MD: Mean disparity
N: *Notarius*
P: *Potamarius*
PC: Principal component
PCA: Principal component analysis
PD: Partial disparity
RAD-seq: Restriction-site associated DNA-sequencing
S: *Sciades*
SNP: Single-nucleotide polymorphisms
TEP: Tropical Eastern Pacific
TPS: Thin-plate spline
