# Supplementary material for: Study of morphological variation of northern Neotropical Ariidae reveals conservatism despite macrohabitat transitions
Source: BMC Evol Biol. 2018 Mar 27;18:38. doi: 10.1186/s12862-018-1152-y (PMC5870521; doi:10.1186/s12862-018-1152-y)
Supplement: Supplementary file 1 — Supporting information for the Methods and Results section. Contains Tables S1 to S4: list of species and number of individuals used in this study, geographic locations, and definitions of geometric morphometric landmarks, and Figure S1 – PC scatterplot with species highlighted. (DOCX 261 kb) [file 12862_2018_1152_MOESM1_ESM.docx]

# Supporting information

# Study of morphological variation of northern Neotropical Ariidae reveals conservatism despite macrohabitat transitions

Madlen Stange^1^, Gabriel Aguirre-Fernández^1^, Walter Salzburger^2^, Marcelo R. Sánchez-Villagra^1^

**Addresses**

1 Palaeontological Institute and Museum, University of Zurich, Karl-Schmid-Strasse 4, 8006 Zurich, Switzerland

2 Zoological Institute, University of Basel, Vesalgasse 1, 4051 Basel, Switzerland

Table S1. List of species and number of specimens used for morphospace analysis.

| **Species** | **Number of specimens** |
| --- | --- |
| *Ariopsis seemanni* (Günther 1864) | 5 |
| *Ariopsis jimenzi* Marceniuk, Acero, Cooke, Betancur R. 2017 | 10 |
| *Bagre bagre* (Linnaeus, 1766) | 5 |
| *Bagre marinus (Mitchill, 1815)* | 17 |
| *Bagre panamensis* (Gill 1863) | 3 |
| *Bagre pinnimaculatus* (Steindachner 1876), called *B. pinnimaculatus* 1 in text | 4 |
| *Bagre aff. pinnimaculatus* (undescribed, reported in Stange et al., 2016 and Stange et al., 2017), called *B. pinnimaculatus* 2 in text | 21 |
| *Cathorops fuerthii* (Steindachner, 1876) | 5 |
| *Cathorops nuchalis* (Günther 1864) | 11 |
| *Cathorops steindachneri* (Gilbert & Starks 1904) | 1 |
| *Cathorops wayuu* Betancur-R., Acero P. & Marceniuk (2012) | 46 |
| *Cathorops tuyra* (Meek & Hildebrand, 1923) | 7 |
| *Notarius biffi* Betancur-R and Acero (2004) | 1 |
| *Notarius cookei* (Acero and Betancur-R 2002) | 7 |
| *Notarius grandicassis* (Valenciennes 1840) | 9 |
| *Notarius planiceps* (Steindachner, 1877) | 1 |
| *Notarius quadriscutis* (Valenciennes, 1840) | 7 |
| *Notarius kessleri* (Steindachner 1876) | 10 |
| *Sciades dowii* (Gill 1863) | 5 |
| *Sciades proops* (Valenciennes, 1840) | 19 |
| *Sciades herzbergii* (Bloch, 1784), called *S. herzbergii* GOV in text | 26 |
| *Sciades aff. herzbergii* (undescribed, reported in Stange et al., 2016 and Stange et al., 2017), called *S. herzbergii* CLA in text | 19 |
| *Sciades parkeri* (Traill 1832) | 1 |
| *Doraops zuloagai* Schultz 1944 | 11 |

Table S2. Geographic locations of sampling sites with GPS coordinates and habitat (marine, brackish, freshwater) attribution.

| **Location** | **Country** | **GPS coordinates** | **Habitat** | **Species** |
| --- | --- | --- | --- | --- |
| Lago de Maracaibo / Isla de Toas | Zulia, Venezuela | 10°57'9.50"N 71°38'49.54"W | brackish | *C. wayuu* |
| Lago de Maracaibo /Isla de San Carlos | Zulia, Venezuela | 10°59'55.1''N 71°36'19.8''W | brackish | *B. bagre* |
| Lago de Maracaibo / Puerto Concha | Zulia, Venezuela | 9°05’46.0”N 71°42´52”W | freshwater | *C. nuchalis* |
| Lago de Maracaibo / Guarico | Zulia, Venezuela | 10°43'52.0''N 71°31'40.2''W | brackish | *C. wayuu* |
| Gulf of Venezuela | Falcon, Venezuela | 11°14'15.3''N 70°30'53.1'' W | marine | *S. proops, S. herzbergii, C. wayuu, B. marinus, B. bagre, N. grandicassis* |
| Clarines | Anzoategui, Venezuela | 10° 3'46.76"N 65°11'5.23"W | brackish | *N. quadriscutis, S. aff. herzbergii, S. proops* |
| Puerto La Cruz | Anzoategui, Venezuela | 10°12'58.63"N 64°38'39.16"W | marine | *C. wayuu, B. marinus S. proops* |
| Ciudad Bolivar | Bolívar, Venezuela | 8°8'51.46" N 63°32'10.68"W | freshwater | *S. parkeri* |
| Pearl Islands / Casaya island | Panama | 8°34'38.64"N 79°3'3.636" W | marine | *A. jimenzi* |
| Puente del Rio Chagres | Panama | 9°11'34.66"N 79°39'9.42"W | freshwater | *C. tuyra* |
| Rio Hato | Panama | 8°20'32.4"N 80°09'56.4"W | brackish | *C. fuerthi, S. dowii* |
| Rio Santa Maria | Panama | 8° 6'20.30"N 80°33'16.06"W | freshwater | *N. cookei, S. dowii* |
| Rio Parita | Panama | 8°01'13.69"N 80°27'11.15"W | brackish | *C. fuerthi* |
| Rio Estero Salado | Panama | 8°10'30.324"N 80°29'35.052" W | brackish | *B. pinnimaculatus, B. panamensis, N. planiceps, N. kessleri, A. seemanni* |
| Rio San Pedro | Panama | 7°50' 59.208"N 81°07' 3.972" W | brackish | *N. kessleri, N. biffi* |
| Puerto Caimito | Panama | 8°52'18.88"N 79°42'32.99"W | marine | *S. dowii* |
| Gulf of Panama | Panama | 8°48'56.55"N 79°22'50.85"W | marine | *B. pinnimaculatus, B. aff pinnimaculatus* |
| Rio El Palmar, Puerto Rico | Zulia, Venezuela | 10°13'32.92"N 71°55'12.93"W | freshwater | *Doraops zuloagai* (Doradidae) |

Table S3. List of museum specimens used for extension of morphospace analysis.

| **catalog no.**  **UMMZ** | **taxonomy used by UMMZ** | **taxonomy used in the present study** | **locality and habitat** |
| --- | --- | --- | --- |
| 196479-1 | *Arius assimilis* | *Ariopsis assimilis^a^* | Mexico, Laguna Bacalur, Caribbean drainage, brackish |
| 197184-3 | *Arius assimilis* | *Ariopsis assimilis^a^* | Guatemala, Rio Dulce, freshwater |
| 197214-1 | *Arius assimilis* | *Ariopsis assimilis^a^* | Guatemala, Rio Nimblaja, freshwater |
| 197214-2 | *Arius assimilis* | *Ariopsis assimilis^a^* | Guatemala, Rio Nimblaja, freshwater |
| 179147-1 | *Arius felis* | *Ariopsis felis^a, b^* | USA, Florida, Bay Gulf of Mexico, marine |
| 179147-2 | *Arius felis* | *Ariopsis felis^a, b^* | USA, Florida, Bay Gulf of Mexico, marine |
| 186995-2 | *Arius felis* | *Ariopsis felis^a, b^* | USA, Florida, Monroe, Indian Key, marine |
| 248826-1 | *Arius felis* | *Ariopsis felis^a, b^* | USA, Florida, Osprey Nest, brackish |
| 198711-1 | *Arius sp.* | *Cathorops aguadulce^b^* | Guatemala, Laguna de Ronpiro, Usumacinta drainage, freshwater |
| 198711-2 | *Arius sp.* | *Cathorops aguadulce^b^* | Guatemala, Laguna de Ronpiro, Usumacinta drainage, freshwater |
| 198712-1 | *Arius aguadulce* | *Cathorops aguadulce^b^* | Mexico, Rio Salinas, Usumacinta drainage, freshwater |
| 143496-1 | *Potamarius nelsoni* | *Potamarius nelsoni* | Guatemala, Rio de la Pasion, Usumacinta drainage, freshwater |
| 198713-2 | *Potamarius nelsoni* | *Potamarius nelsoni* | Guatemala, Rio de la Pasion, Usumacinta drainage, freshwater |
| 198721 | *Potamarius nelsoni* | *Potamarius nelsoni* | Guatemala, Rio de la Pasion, Usumacinta drainage, freshwater |
| 188048-1 | *Potamarius usumacintae* | *Potamarius usumacintae* | Guatemala, Rio de la Pasion, Usumacinta drainage, freshwater |
| 190074-2 | *Potamarius usumacintae* | *Potamarius usumacintae* | Guatemala, Rio de la Pasion, Usumacinta drainage, freshwater |
| 190074-1 | *Potamarius usumacintae* | *Potamarius usumacintae* | Guatemala, Rio de la Pasion, Usumacinta drainage, freshwater |
| 198716 | *Potamarius usumacintae* | *Potamarius usumacintae* | Guatemala, Rio de la Pasion, Usumacinta drainage, freshwater |
| 198715 | *Potamarius usumacintae* | *Potamarius usumacintae* | Guatemala, Rio de la Pasion, Usumacinta drainage, freshwater |

UMMZ: University of Michigan Museum of Zoology

^a^ [1], ^b^ [2]

Table S4. Definition of the 17 landmarks used to study the neurocranial morphology and shape changes in ariid species from freshwater, brackish, and marine habitat.

| **Landmark #** | **defined by** |
| --- | --- |
| **1** | most lateral point of left posttemporosupracleithrum |
| **2** | lateral suture of left sphenotic and frontal |
| **3** | most lateral point of left lateral ethmoid |
| **4** | medial curvature of left portion of mesethmoid |
| **5** | most lateral point of left portion of mesethmoid |
| **6** | most lateral point of right portion of mesethmoid |
| **7** | medial curvature of right portion of mesethmoid |
| **8** | most lateral point of right lateral ethmoid |
| **9** | lateral suture of right pterotic and sphenotic |
| **10** | most lateral point of right posttemporosupracleithrum |
| **11** | sutures meeting of left sphenotic, pterotic and supraoccipital |
| **12** | sutures meeting of left and right frontal meeting supraoccipital |
| **13** | sutures meeting of right sphenotic, pterotic and supraoccipital |
| **14** | caudal part of supraoccipital process |
| **15** | left opening of visual nerve |
| **16** | right opening of visual nerve |
| **17** | opening of aortic canal |


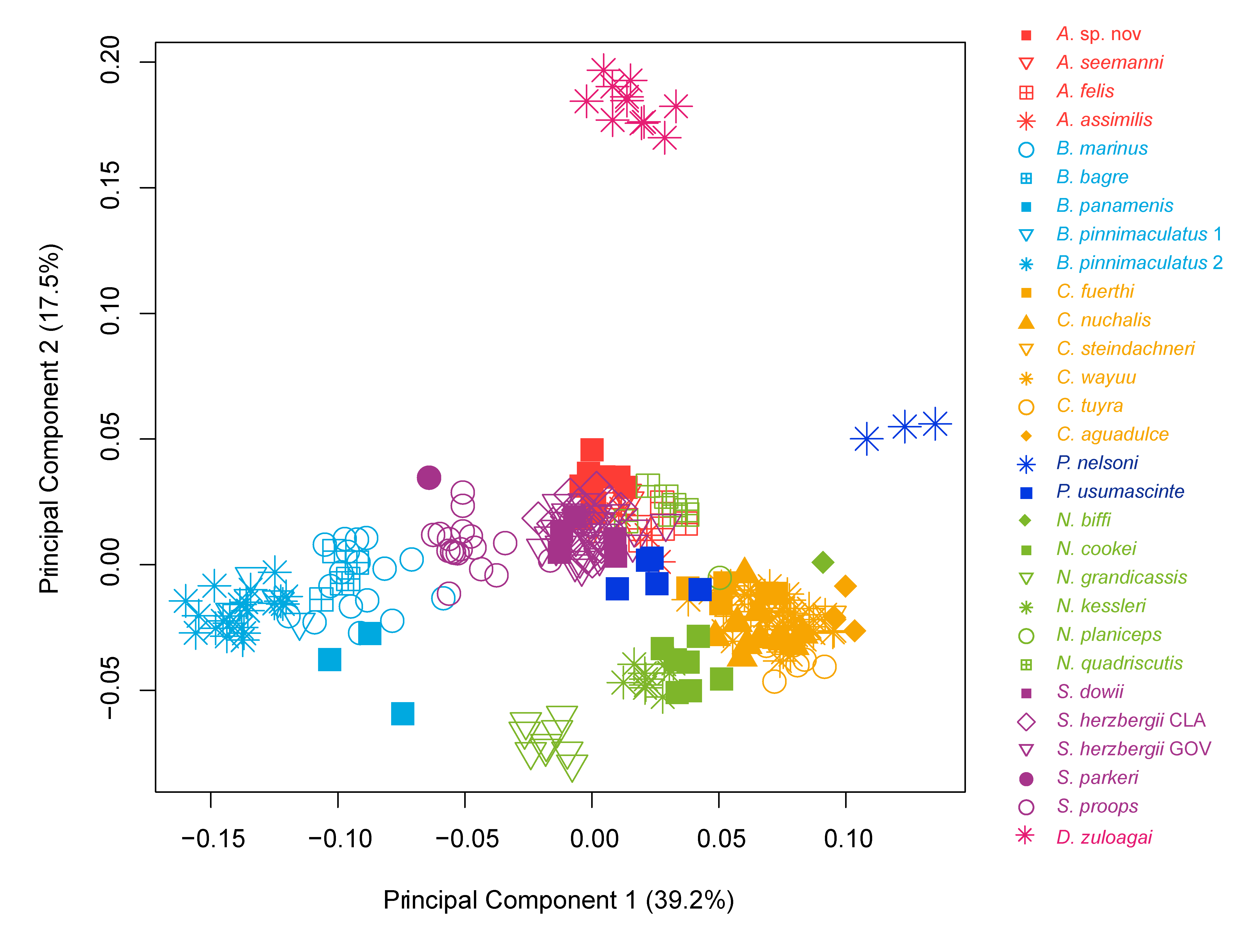


Figure S1. PC1-PC2 morphospace plot of 28 northern Neotropical ariid and one doradoid species. Genus affinity highlighted by colour, species affinity highlighted by symbols. This plot is identical to Figure 3 but identifies species.

# References

1. Betancur-R. R, Acero P. A, Bermingham E, Cooke R. Systematics and biogeography of New World sea catfishes (Siluriformes: Ariidae) as inferred from mitochondrial, nuclear, and morphological evidence. Mol. Phylogenet. Evol. 2007;45:339–57.

2. Betancur-R. R. Molecular phylogenetics and evolutionary history of ariid catfishes revisited: a comprehensive sampling. BMC Evol. Biol. 2009;9:175.
